# Unraveling the Complex Relationship Between Gastroesophageal Reflux Disease, Lifestyle Factors, and Interstitial Lung Disease: Insights From Two-Sample Mendelian Randomization Analyses

**DOI:** 10.7759/cureus.51220

**Published:** 2023-12-28

**Authors:** Mohammad A Jareebi, Nawaf F Gharawi, Mohammed O Shami, Alhassan M Kariri, Tariq F Hakami, Nasser M Alamer, Khalid M Alhazmi, Ali M Kariri, Abdurahman H Darbashi, Anwar M Kuriri, Ali Mohammed Someili

**Affiliations:** 1 Community and Family Medicine, Jazan University, Jazan, SAU; 2 Faculty of Medicine, Jazan University, Jazan, SAU; 3 Pediatrics, Ministry of Health, General Directorate of Health Affairs in Jazan, Jazan, SAU; 4 Internal Medicine, Ministry of Health, General Directorate of Health Affairs in Jazan, Jazan, SAU; 5 ‏Internal Medicine, Ministry of Health, General Directorate of Health Affairs in Jazan, Jazan, SAU; 6 Internal Medicine and Pulmonology, Ministry of Health, General Directorate of Health Affairs in Jazan, Jazan, SAU; 7 Internal Medicine and Pulmonology, King Fahad Central Hospital, Jazan, SAU; 8 Internal Medicine and Gastroenterology, Faculty of Medicine, Jazan University, Jazan, SAU

**Keywords:** general internal medicine, mendelian randomization, lifestyle factors, gerd, interstitial lung disease

## Abstract

Background

Although the cause of interstitial lung disease (ILD) remains uncertain, it is believed to be a combination of genetic and non-inherited factors, such as smoking and diet. This research aims to evaluate the impact of gastroesophageal reflux disease (GERD) and other modifiable risk factors on the likelihood of developing ILD by utilizing two-sample Mendelian randomization.

Methodology

The research utilized publicly accessible single-nucleotide polymorphisms (SNPs) that were deemed significant on a genome-wide scale. These SNPs were chosen from prior studies conducted by various consortia. The study examined GERD and a wide range of smoking habits, including the age at which individuals started smoking, the intensity of their smoking, and whether their mothers smoked. Additionally, the study considered other relevant risk factors such as key dietary factors, coffee consumption, body mass index (BMI), and physical activity. The study focused on self-reported ILD as its outcome measure. The genetic information for ILD was sourced from the FinnGen and UK Biobank (UKB) cohorts.

Results

The study encompassed a wide range of sample sizes, varying from 64,949 to 632,802, for each risk factor collected from multiple consortia. In total, 593 SNPs were included for all risk factors. The findings revealed significant associations between genetically estimated GERD, dietary factors, BMI, and the risk of ILD within the FinnGen consortium. The odds ratios (ORs) indicated an increase in the risk of ILD per unit of GERD (OR = 1.17, p = 0.001), smoking initiation (OR = 1.10, p < 0.05), BMI (OR = 1.15, p = 0.006), and low-density lipoprotein (LDL) (OR = 1.10, p = 0.02). On the other hand, there was a decrease in the risk of ILD per unit increase in coffee intake (OR = 0.64, p = 0.01) and physical activity (OR = 0.79, p=0.03). Additionally, the results demonstrated a significant association between genetically estimated GERD (OR = 1.01, p < 0.05), coffee intake (OR = 1.14, p=0.03), and high-density lipoproteins (HDL) (OR = 1.01, p=0.04) and increased risk of ILD specifically within the UKB.

Conclusions

This research indicates that the development of ILDs may be causally associated with GERD and various factors such as coffee intake, smoking, BMI, physical activity, LDL, and HDL These results hold great importance in terms of devising effective strategies for the treatment and prevention of ILDs.

## Introduction

Interstitial lung diseases (ILDs) encompass a diverse group of non-malignant, non-infectious conditions affecting the lower respiratory tract. These conditions are characterized by inflammation and fibrosis in the alveoli and interstitium, as observed through histological examination [1]. Among the ILDs, idiopathic pulmonary fibrosis (IPF) and systemic sclerosis (SSc) have been associated with gastroesophageal reflux disease (GERD). GERD, a potential risk factor for microaspiration, is also considered a potential risk factor for IPF [2]. However, interpreting epidemiological data on the association between GERD and IPF has been challenging due to methodological limitations. GERD is characterized by the non-physiological aspiration of gut contents, which can lead to troublesome symptoms and complications such as esophagitis. Observational studies have suggested a potential association between GERD and IPF, but it is unclear whether GERD causes an increased risk of IPF [2]. Other factors, such as smoking, may be confounding the observed association. Additionally, IPF itself could be causing an increased risk of GERD due to reduced lung compliance and negative intrathoracic pressures [3]. To investigate the potential causal relationship between GERD and IPF, a Mendelian randomization (MR) approach using genetic variants as proxies could provide valuable indirect evidence. Lifestyle factors have been implicated in both GERD and ILDs. In the case of ILD, smoking is known to damage the lungs or worsen lung damage from other factors [4]. Some forms of ILDs occur more frequently in people with a history of smoking. Moreover, active smoking may worsen some ILDs. Lifestyle factors such as smoking, eating certain food triggers, and eating habits can worsen GERD.

The objective of this study is two-fold. The first objective is to assess the causal relationship between GERD and the development of ILD. The second objective is to explore the causal impact of modifiable risk factors such as dietary habits, smoking, and lipid biomarkers on the risk of ILD. It is hypothesized that there is a causal relationship between GERD and the development of ILD. Furthermore, it is hypothesized that smoking, lipid biomarkers, and dietary habits are modifiable risk factors for ILD.

## Materials and methods

Mendelian randomization and genetic instrumentation

MR is a technique used to establish a causal relationship between a modifiable exposure and an outcome by utilizing genetic variants found in observational data [5]. These genetic variants, referred to as single-nucleotide polymorphisms (SNPs), naturally differ among individuals [6]. SNPs are selected through genome-wide association studies (GWAS) based on their strong association (P=5x10-8) with specific characteristics, such as GERD, smoking status, and BMI [7]. In MR analysis, these SNPs serve as proxies for the exposure, as long as they are not associated with other variables (non-pleiotropic) and specifically relate to that particular trait [5]. MR can be conducted using either a one-sample or two-sample approach to establish causal inferences [8]. In one-sample MR, both the exposure and outcome data are obtained from the same cohort. On the other hand, two-sample MR combines exposure data from one population with outcome data from another [8]. This study employed the two-sample approach.

Cohorts and data sources

The UK Biobank (UKB) is a large-scale research project that includes approximately 502,000 individuals who were evaluated at 22 assessment facilities across England, Scotland, and Wales between 2006 and 2010. The evaluation process involved a comprehensive assessment of medical, psychological, and anthropometric criteria, including self-reported data on conditions such as ILD [8]. On the other hand, FinnGen, a genetic research initiative based in Finland, aims to collect genetic information from 500,000 Finnish individuals. Its main objective is to explore the relationship between genetic factors and various diseases. Launched in 2017, this project is expected to be completed by 2025, with over 200,000 Finnish individuals already contributing their genetic data to support the study [9]. This study utilized publicly available summary-level information obtained from these combined datasets. In this study, the researchers focused on investigating the potential causal role of GERD in the development of ILD. Additionally, they examined various other potential risk factors that could potentially influence the development of ILD. These factors included age at smoking initiation, smoking intensity, historical maternal smoking, vitamin D levels, cheese intake, salad intake, coffee intake, body mass index (BMI), lipid biomarkers (cholesterol, low-density lipoprotein (LDL), triglycerides (TG), and high-density lipoproteins (HDL)), and self-reported physical activity.

SNP selection

The researchers identified relevant variables by utilizing genome-wide significant SNPs from various consortia. These include the UKB [10], GWAS and Sequencing Consortium of Alcohol and Nicotine Use (GSCAN) [11], Genetic Investigation of Anthropometric Traits (GIANT) [12], Global Lipids Genetics Consortium (GLGC) [13], and Klimentidis et al. (2018) [14]. These SNPs are associated with specific traits and have been determined through GWAS with a significance threshold of P<5x10-8 [7].

**Figure 1:**
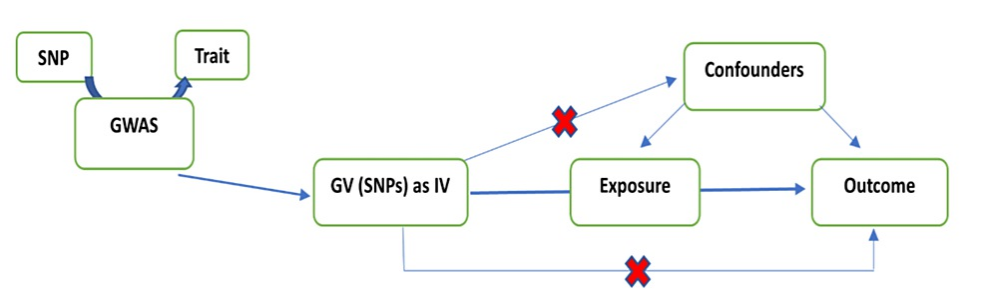
Mendelian randomization design Mendelian randomization design adopted from Jareebi (2022) [28]. GWAS: genome-wide association studies; SNPs: single-nucleotide polymorphisms

The study utilized a distinct set of SNPs that are widely used in the literature [15-17] for each exposure. Specifically, 80 SNPs for GERD [18], 93 SNPs for smoking initiation [19], six SNPs for smoking intensity [20], seven SNPs for maternal smoking [21], 177 SNPs for vitamin D [22], 65 SNPs for cheese intake [23], 22 SNPs for salad intake [24], three SNPs for coffee intake [25], 79 SNPs for BMI [12], seven SNPs for physical activity [26], and 46, 41, 55, and 89 SNPs for cholesterol, LDL, TG, and HDL, respectively [27]. Figure 1 illustrates the MR design.

Statistical analysis and genetic data integration

The main aim of this study was to evaluate the link between GERD and ILD, which is a binary outcome. Genetic information on ILD was sourced from two different databases, namely, UKB and FinnGen [9,10]. Following data harmonization, which involves aligning and standardizing the reporting of genetic associations to ensure that the effect alleles for exposures and outcomes are consistently expressed per additional copy of the same allele, we analyzed a set of SNPs for each exposure factor about ILD.

To conduct MR and sensitivity analyses, we utilized the TwoSampleMR package in R software (version 4.2.3). The analysis involved collecting genetic data for the exposures and their corresponding outcomes. To ensure compatibility, data harmonization was performed to match alleles across two separate datasets, facilitating MR analysis. Separate MR analyses were performed for migraine using data from both the UKB and FinnGen consortia. A significance level of p < 0.05 was applied to all MR analyses, with a primary focus on the inverse variance weighted (IVW) method. Additionally, we employed more restrictive MR measures, including MR-Egger, which considers increased pleiotropy, to identify any potential deviations from IVW results.

Ethical approval

No ethical approval was required as this study consisted of publicly accessible summary statistics from a GWAS dataset. Furthermore, the original study was done by relevant ethical regulations and guidelines, and appropriate informed consent was obtained from the study participants.

## Results

Genetic profiling of risk factors

A total of 593 SNPs were assessed across various risk factors, with each factor evaluated using a range of three to 93 SNP variants. These genetic markers were obtained from different consortia, each with varying sample sizes, spanning from 64,949 to 632,802 individuals per risk factor (see Table 1 for details).

**Table 1:** Overview of genetic risk factors: a brief summary. BMI: body mass index; GIANT: The Genetic Investigation of ANthropometric Traits; GLGC: The Global Lipids Genetics Consortium; GSCAN: GWAS & Sequencing Consortium of Alcohol and Nicotine Use; GWAS: genome-wide association studies; HDL: high-density lipoproteins; LDL: low-density lipoproteins; SNPs: single-nucleotide polymorphisms; TG: triglycerides; UKB: UK Biobank; GERD: gastroesophageal reflux disease

Exposure	Number of SNPs	Sample size	Population (consortium)
GERD	80	602,604	Ong JS
Smoking initiation	93	632,802	GSCAN
Smoking intensity	6	108,946	Within the family GWAS consortium
Maternal smoking	7	289,727	UKB
Cheese intake	65	451,486	UKB
Salad intake	22	462,933	UKB
Coffee intake	3	64,949	UKB
Vitamin D	177	44,1291	Manousaki et al. [22]
BMI	79	339,152	GIANT
Physical activity	7	261,055	Klimentidis et al. [14]
Cholesterol	46	92,260	GLGC
LDL	41	83,193	GLGC
TG	55	177,861	GLGC
HDL	89	187,167	GLGC

ILD genetic characteristics

The ILD data were collected from two separate population groups, specifically the UKB and FinnGen. The UKB cohort includes 7,637 participants, with approximately 96 individuals diagnosed with ILD. The FinnGen sample comprises 218,792 participants, of which around 21,806 have reported cases of ILD.

ILD risk in the FinnGen cohort

**Figure 2:**
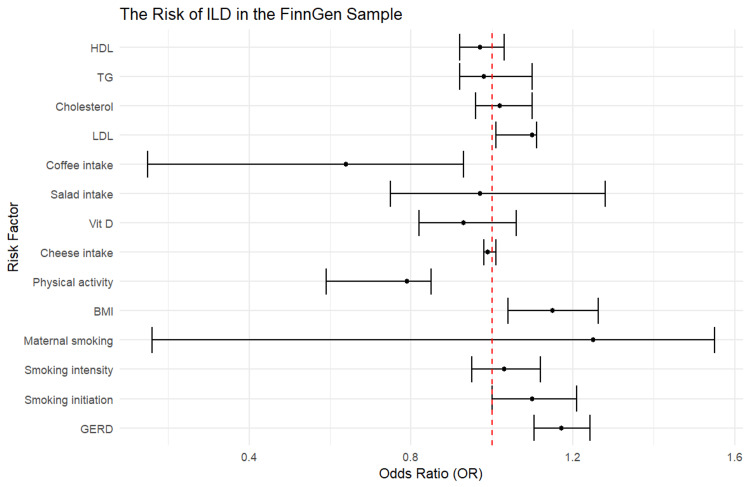
The risk of ILD in the FinnGen sample. BMI: body mass index; HDL: high-density lipoproteins; LDL: low-density lipoproteins; SNPs: single-nucleotide polymorphisms; TG: triglycerides; GERD: gastroesophageal reflux disease; FinnGen: Finnish Health Research Environment for Genomic Research; ILD: interstitial lung disease

The findings of the two-sample MR analysis in the FinnGen sample revealed a significant positive relationship between genetically estimated GERD (odds ratio (OR) = 1.17, 95% confidence interval (CI) = 1.10-1.24, p < 0.001), early smoking initiation (by one year) (OR = 1.10, 95% CI = 1.001-1.21, p < 0.05), one higher standard deviation (SD) of BMI (OR = 1.15, 95% CI = 1.04-1.26, p = 0.006), and one SD higher LDL (OR = 1.10, 95% CI = 1.01-1.11, p = 0.02), all of which were associated with the development of ILD. Conversely, higher physical activity and coffee intake were associated with decreased risk of ILD (OR = 0.64, 95% CI = 0.15-0.93, p = 0.012 and OR = 0.79, 95% CI = 0.59-0.85, p = 0.03, respectively) (Figure 2, Table 2).

**Table 2:** Overview of ILD findings in the FinnGen cohort. BMI: body mass index; HDL: high-density lipoproteins; LDL: low-density lipoproteins; SNPs: single-nucleotide polymorphisms; TG: triglycerides; GERD: gastroesophageal reflux disease; FinnGen: Finnish Health Research Environment for Genomic Research; ILD: interstitial lung disease

Risk factor	OR (95% CI)	P-value
GERD	1.172 (1.104–1.242)	0.001124
Smoking initiation	1.10 (1.001–1.21)	<0.05
Smoking intensity	1.03 (0.95–1.12)	0.453
Maternal smoking	1.25 (0.16–1.55)	0.680
BMI	1.15 (1.04–1.263)	0.006
Physical activity	0.79 (0.59–0.85)	0.03
Cheese intake	0.99 (0.98–1.01)	0.799
Vitamin D	0.93 (0.82–1.06)	0.216
Salad intake	0.97 (0.75–1.28)	0.942
Coffee intake	0.64 (0.15–0.93)	0.012
LDL	1.10 (1.01–1.11)	0.022
Cholesterol	1.02 (0.96–1.10)	0.388
TG	0.98 (0.92–1.10)	0.671
HDL	0.97 (0.92–1.03)	0.350

ILD risk in the UKB cohort

**Figure 3:**
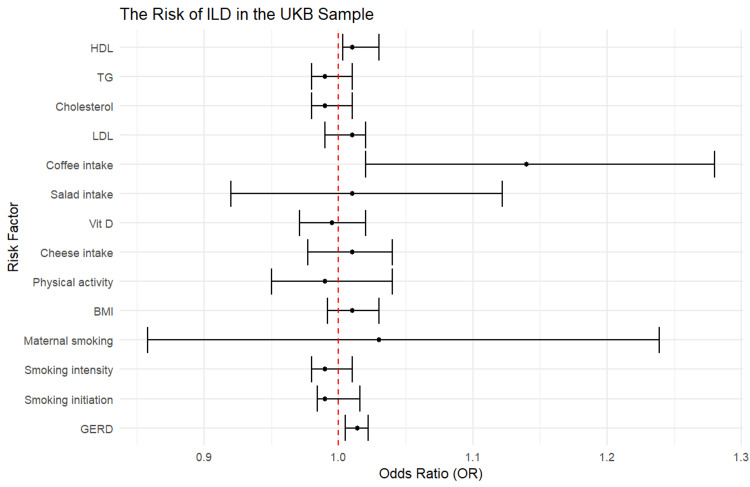
The risk of ILD in the UKB sample. BMI: body mass index; HDL: high-density lipoproteins; LDL: low-density lipoproteins; SNPs: single-nucleotide polymorphisms; TG: triglycerides; GERD: gastroesophageal reflux disease; UKB: UK Biobank; ILD: interstitial lung disease

In the UKB consortium, the analysis showed similar results regarding the association between genetically estimated GERD and an increased risk of ILD (OR = 1.01, 95% CI = 1.005-1.02, p < 0.05). Additionally, in contrast to FinnGen, genetically estimated coffee consumption was positively associated with the risk of ILD in the UKB cohort (OR = 1.14, 95% CI = 1.02-1.28, p = 0.03). Furthermore, one SD higher levels of HDL were associated with an increased risk of ILD (OR = 1.01, 95% CI = 1.003-1.03, p = 0.043) (Figure 3, Table 3).

**Table 3:** Overview of ILD findings in the UKB cohort. BMI: body mass index; HDL: high-density lipoproteins; LDL: low-density lipoproteins; SNPs: single-nucleotide polymorphisms; TG: triglycerides; GERD: gastroesophageal reflux disease; UKB: UK Biobank; ILD: interstitial lung disease

Risk factor	OR (95% CI)	P-value
GERD	1.014 (1.005–1.022)	<0.05
Smoking initiation	0.99 (0.984–1.016)	0.973
Smoking intensity	0.99 (0.98–1.01)	0.465
Maternal smoking	1.03 (0.858–1.239)	0.721
BMI	1.01 (0.992–1.03)	0.278
Physical activity	0.99 (0.95–1.04)	0.991
Cheese intake	1.01 (0.977–1.04)	0.561
Vitamin D	0.995 (0.971–1.02)	0.725
Salad intake	1.01 (0.92–1.122)	0.741
Coffee intake	1.14 (1.02–1.28)	0.033
Cholesterol	1.01 (0.99–1.02)	0.245
LDL	0.99 (0.98–1.01)	0.583
TG	0.99 (0.98–1.01)	0.405
HDL	1.01 (1.003–1.03)	0.043

## Discussion

The two goals of the study were to establish a causal relationship between GERD and ILD and to identify the causal impact of modifiable factors. The significant ILD risk factors in the FinnGen cohort were GERD, smoking initiation, BMI, physical activity, coffee intake, and LDL. In the UKB cohort, the significant risk factors for ILD were GERD, coffee intake, and HDL. Therefore, in both samples, GERD was associated with a statistically significant higher risk of ILD. This finding aligns with those from previous studies which suggested that GERD is a risk factor for ILD [2,3]. GERD may involve other organs such as the throat and the lungs resulting in atypical symptoms such as asthma, dental erosion, and chronic cough [2]. Theoretically, GERD may cause chronic reflux, microaspiration, alveolar inflammation, and fibrotic remodeling which contribute to ILD. In mice, the presence of gastric content in the lungs has been shown to induce pulmonary fibrosis. Bile acids and acidic reflux could also induce fibroblast proliferation and transforming growth factor beta production in cultured tissue [29].

The lifestyle factors that may modify the causal impact of GERD are coffee intake, smoking initiation, BMI, physical activity, LDL, and HDL. Coffee intake had mixed effects on ILD risk. In the FinnGEn cohort, it reduced the risk of ILD, while in the UKB cohort, it increased the risk of ILD. Due to the differences in the direction of the impact across the two samples, there is a need for further research on the role of coffee in the risk of ILD. Most of the identified modifiable factors including BMI, physical activity, coffee intake, and smoking may be risk factors for ILD in some people. Individuals with high BMI and those who do not engage in adequate physical activity are at greater risk of GERD, which, in turn, could influence their risk of ILD [30].

Despite the robustness and validity of our MR findings, this study does have some limitations. First, it is important to note that the GWAS data used for lifestyle factors and GERD in this study were obtained from European populations. Therefore, it is crucial to exercise caution when generalizing our findings to other populations. Second, not all possible confounders of the relationship between GERD and ILD were considered in the study.

GERD should be considered in future investigations of ILD risk, and there should be a renewed interest in exploring it as a potential therapeutic target. The effectiveness of treating GERD in ILD is not well established. In practice, the management of GERD in ILD using antacids is not recommended due to the increased risk of bacterial infections. This study goes some way in addressing the uncertainty that exists regarding the benefits of managing GERD to prevent ILD and its management. Thus, there is a need for well-designed randomized controlled trials in this area to address this research and practice gap.

The prevention and management of ILD should also emphasize the importance of managing coffee intake, smoking, BMI, physical activity, LDL, and HDL. Moderate use of coffee, smoking cessation, regular physical activity, healthy BMI, and good lipid profile are all important considerations for healthy living. However, the evidence from the study suggests that focusing on these elements could help lower the risk of ILD. There is a need for further research into how each of these modifiable risk factors contributes to the risk of ILD and specific medical and nursing interventions that can be implemented to improve them in persons who are at risk of ILD.

## Conclusions

The purpose of the study was to determine the causal relationship between GERD and ILD. Using MR and secondary data from two cohorts, the study revealed that GERD is associated with an increased risk of ILD. Moreover, coffee intake, smoking, BMI, physical activity, LDL, and HDL are potentially modifiable factors that influence the contributions of GERD to ILD. There is a need for further research into the role of coffee intake as the findings from the two cohorts suggest different directions of influence. Moreover, research should focus on the management of GERD among persons with ILD and those at risk of the condition.
